# Supplementary material for: The prognostic value of tumor length to resectable esophageal squamous cell carcinoma: a retrospective study
Source: PeerJ. 2017 Jan 31;5:e2943. doi: 10.7717/peerj.2943 (PMC5289103; doi:10.7717/peerj.2943)
Supplement: Table S1 — The result of model diagnostics on each covariate and the global test for the model as a whole. [file peerj-05-2943-s001.pdf]

	Rho	chisq	*p*
T	0.0383	0.257	0.6119
N	-0.0617	0.65	0.4199
Tumor length	-0.1335	3.488	0.0618
GLOBAL	NA	4.834	0.1844

Note: The significance level: 0.05
