# Supplementary figures and images for: The prognostic value of tumor length to resectable esophageal squamous cell carcinoma: a retrospective study
Source: PeerJ. 2017 Jan 31;5:e2943. doi: 10.7717/peerj.2943 (PMC5289103; doi:10.7717/peerj.2943)

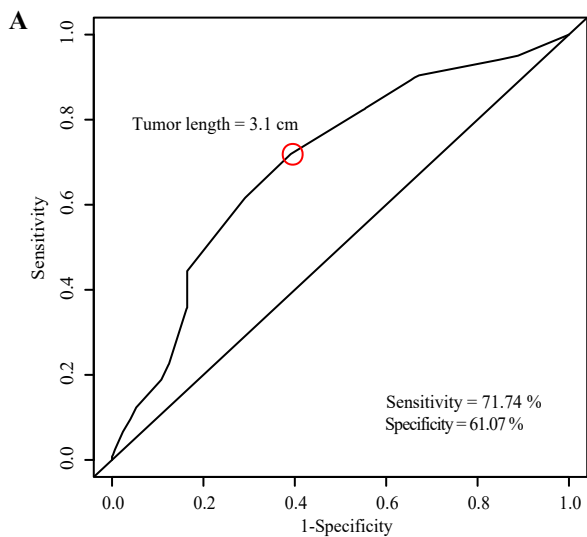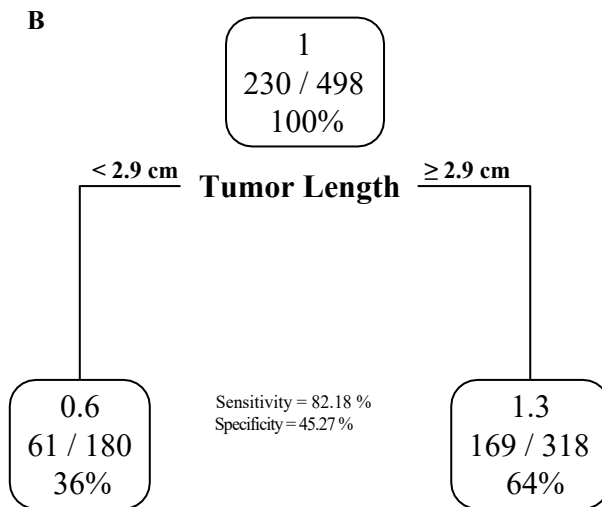

Supplement: Figure S1 — (A) Time-dependent ROC curves to predict ESCC survival. The optimal cut-off point was indicated by the red circle with the highest Youden index (sensitivity + specificity − 1). (B) Regression tree for survival. Node 0 (the upper square of the tree) was known as the root node, which indicated the starting point of the tree construction and included all the patients. This node was divided based on the tumor length of 2.9 cm. Tumor length was correlated with an increasing hazard ratio (HR) for death between the tumor length <2.9 cm with a HR 0.6 and the tumor length ≥2.9 cm with a HR 1.3. The numbers in the node represented HR (top), the ratio of survival vs. total in each category (middle) and the percentage of observations (bottom), respectively. [file peerj-05-2943-s003.pdf]
